# Reversal of pulmonary arterial hypertension and neointimal formation by kinin B1 receptor blockade

**DOI:** 10.1186/s12931-021-01875-w

**Published:** 2021-10-30

**Authors:** Dileep Reddy Rampa, Priya Murugesan, Honglu Chao, Huiying Feng, Wenxin Dai, Dongwon Lee, Anton Pekcec, Henri Doods, Dongmei Wu

**Affiliations:** 1 Department of Bio-Nanotechnology and Bio-Convergence Engineering, Chonbuk National University, Jeonju, South Korea; 2 Department of Neurosurgery, the First Affiliated Hospital of Nanjing Medical University, Nanjing, China; 3 Research Beyond Borders, Boehringer Ingelheim Pharma GmbH & Co. KG, Biberach, Germany; 4 Department of Research, Mount Sinai Medical Center, Miami Beach, FL USA

**Keywords:** Inflammation, Macrophage, Neointimal formation, Pulmonary arterial remodeling, Right heart hypertrophy

## Abstract

**Background:**

This study examined whether BI113823, a novel selective kinin B1 receptor antagonist can reverse established pulmonary arterial hypertension (PAH), prevent right heart failure and death, which is critical for clinical translation.

**Methods:**

Left pneumonectomized male Wistar rats were injected with monocrotaline to induce PAH. Three weeks later, when PAH was well established, the rats received daily treatment of BI113823 or vehicle for 3 weeks.

**Results:**

Treatment with BI113823 from day 21 to day 42 after monocrotaline injection reversed established PAH as shown by normalized values of mean pulmonary arterial pressure (mPAP). BI113823 therapy reversed pulmonary vascular remodeling, pulmonary arterial neointimal formation, and heart and lung fibrosis, reduced right ventricular pressure, right heart hypertrophy, improved cardiac output, and prevented right heart failure and death. Treatment with BI113823 reduced TNF-α and IL-1β, and macrophages recruitment in bronchoalveolar lavage, reduced CD-68 positive macrophages and expression of proliferating cell nuclear antigen (PCNA) in the perivascular areas, and reduced expression of iNOS, B1 receptors, matrix metalloproteinase (MMP)-2 and MMP-9 proteins, and the phosphorylation of ERK1/2 and AKT in lung. Treatment with BI113823 reduced mRNA expression of ANP, BNP, βMHC, CGTF, collange-I and IV in right heart, compared to vehicle treated controls. In human monocytes cultures, BI113823 reduced LPS-induced TNF-α production, MMP-2 and MMP-9 expression, and reduced TNF-α-induced monocyte migration.

**Conclusions:**

We conclude that BI113823 reverses preexisting severe experimental pulmonary hypertension via inhibition of macrophage infiltration, cytokine production, as well as down regulation of matrix metalloproteinase proteins.

## Background

Pulmonary arterial hypertension (PAH) pathobiology involves pulmonary arterial remodeling, vasoconstriction and in situ thrombosis, leading to enhanced pulmonary vascular resistance and pressure, to right heart failure and death [1–3]. Inflammation is a prominent pathologic feature in PAH [2–7]. Infiltration of inflammatory cells, including macrophages, are found in pulmonary perivascular spaces and around the plexiform lesions in lung biopsies from patients with PAH [6, 7]. Proinflammatory mediators, such as TNF-α, IL-1β, and IL-6 are elevated in the serum of patients with PAH and can predict the survival of patients with idiopathic PAH [2, 3, 5–7]. Therefore, a better understanding of inflammatory pathways and their role in the pathogenesis of PAH may lead to targeted therapeutic approaches [2–7].

Kinin B1 receptors are involved in a variety of pathological processes of inflammation [8–13]. Kinins are proinflammatory peptides that exert a variety of biological actions via activation of two receptor subtypes, B1 and B2 [8, 9]. Kinin B1 receptors are normally weakly expressed, but are strongly upregulated following injury or exposure to pro-inflammatory agents such as cytokines and endotoxins, while the B2 receptors are expressed constitutively [8, 9]. Kinin B2 receptors activation is thought to mediate most of the beneficial cardiovascular effects of kinins, as well as the beneficial effects of angiotensin 1 converting enzyme inhibitors [8, 9]. In contrast, kinin B1 receptors are involved in diverse pathological processes including inflammation, platelet activation, smooth muscle contraction, increased vascular permeability, edema, pain, cytokine and chemokine release, cell proliferation, and tissue remodeling [8–13], responses that are key components of PAH.

We have identified that kinin B1 receptors play important roles in the pathogenesis of PAH and vascular remodeling [14]. Kinin B1 receptor expression is increased in an experimental model of PAH in rats [14], as well as on foamy macrophages within thickened intimal plaques in patients with atheromatous disease [15]. It is important to note that unlike B2 receptors, kinin B1 receptors are not rapidly internalized and desensitized [10, 16]. Thus kinin B1 receptor-induced responses are more persistent and could contribute to long term pathological changes such as those seen in PAH [10, 16]. Therefore, kinin B1 receptors could represent a novel therapeutic target in chronic inflammatory processes leading to these events [10, 16]. We have shown that early treatment with a selective kinin B1 receptor antagonist BI113823 reduced lung inflammation, prevented the development of PAH and vascular remodeling [14]. PAH is often not recognized until the disease is relatively advanced [1]. Therefore, finding a drug that is effective when administered after established PAH would be of major significance. The present study was designed to examine whether treatment with a small molecule orally-active, non-peptide B1 receptor antagonist BI113823 can rescue preexisting PAH and vascular remodeling, and prevent right heart failure and death in rats. The selection of animal models is also critical to the discovering novel agents for the treatment of PAH. The present study utilized a two “hits” pneumonectomy plus monocrotaline induced PAH model which shows histological evidence of inflammation, and develops severe pulmonary hypertension and vascular remodeling [17, 18]. This model has also been used by many investigators to test new drug treatments on attenuating the development of PH, reversing established disease or promoting survival [14, 17–19]. The cellular mechanisms underlying the protection afforded by kinin B1 receptor inhibition were also examined in animals and in human monocytic cells.

## Materials and methods

These animal studies were approved by the Institutional Animal Care and Use Committee at Chonbuk National University, and the National Institutes of Health (NIH) Guide for the Care and Use of Laboratory Animals. A total of 55 male, 10-week-old Wistar rats, weighing between 250 and 300 g were studied.

### MCT-induced pulmonary hypertension in left pneumonectomized rats

Experimental model of PAH and vascular remodeling was induced by monocrotaline–challenge in left pneumonectomized rats as previously described [14]. Briefly, the rats were anesthetized with intramuscular injections of ketamine (80 mg kg^−1^) and xylazine (10 mg kg^−1^) before undergoing left pneumonectomy. On week later, rats were injected subcutaneously in the right hind limb with monocrotaline (MCT) (60 mg/kg, WAKO, Japan). Three weeks after MCT injection, these rats were then randomly assigned to receive treatment with vehicle (0.5% Natrosol + 0.01% TWEEN 80, p.o., b.i.d., n = 8), or with BI113823 (selective B1 receptor antagonist, 30 mg/kg, p.o., b.i.d., n = 8) for 3 weeks. Additional six animals was used to confirm the development of severe PAH and vascular remodeling at 3 weeks after MCT injection.

At the end of experiment, rats were anesthetized before a pulmonary arterial catheter was inserted through the right internal jugular vein. The right atrial pressure, right ventricular systolic blood pressure (RVSP), and pulmonary arterial pressure (PAP) were recorded using a Powerlab data acquisition system (ADInstruments Inc., CO). Next, another 2F miniaturized combined conductance catheter-micro-manometer (Model SPR-838, Millar instruments, Houston, TX) was inserted into the carotid artery to obtain the arterial pressure. Then, this catheter-micro-manometer was advanced into the left ventricle to record the left ventricular pressure and its first derivative (± dp/dp max).

Bronchoalveolar lavage (BAL) was collected through a 14-gauge angiocatheter. Lavages were collected twice after 2.5 ml of sterile PBS was infused into the rat’s lung. A standard hemocytometer was used for BAL cell counting. Differential cell counts were performed on Giemsa-wright stained (Microscopy Hemacolor-Merck; Germany) cytospin preparations. Lung and heart tissues were collected and weighed. Liquid nitrogen was used to snap freeze one set of the tissues, while other tissues sets were fixed in buffered formalin for histopathological examination. The BAL protein concentration was determined using a Smart BCA Assay Kit (Intron Biotechnology Inc. South Korea). Enzyme immunoassay kits for rat interleukin 1-beta (IL-1β) (R & D Systems, Minneapolis, MN) and tumor necrosis factor (TNF)-α (BioLegend, San Diego, CA) were used to determine the concentrations of these mediators in BAL fluid and plasma. Neutrophil accumulation in the lung was measured by determining myeloperoxidase (MPO) activity as previously described [13].

### Histological analysis

As previously described [14], standard histopathological procedures were used to prepare 5-μm-thick sections. The sections were deparaffinized and stained with hematoxylin and eosin (Sigma, St. Louis, MO, USA), elastic van gieson (EVG, Sigma) and with Masson-Trichrome (American Master Tech Scientific, Inc. Lodi, CA, USA). The lungs were examined with light microscopy for morphological alterations. All of the analysis was blinded. Parameters of vascular remodeling were performed using Image J software. The pulmonary arterial medial wall thickness was calculated as % wall thickness = (wall thickness × 2/external diameter) × 100. The severity of neointimal formation was scored according to methods previously described [17]. In this scoring system: 0 = the absence of neointimal lesion; 1 = less than 50% luminal occlusion; 2 = greater than 50% luminal occlusion. The average score of 50 vessels was obtained for each animal.

### Immunohistochemical analysis

For IHC analysis, 5-μm-lung sections were deparaffinized, hydrated and incubated in 10 mM sodium citrate buffer at 99 °C for 20 min for antigen retrieval. Sections were incubated overnight with a primary antibody to one of the following antigens: kinin B1 receptor, kinin B2 receptor, iNOS, CD68, proliferating cell nuclear antigen (PCNA), matrix metalloproteinase (MMP)-9 (all from Santa Cruz Biotechnology, Santa Cruz, CA), MMP-2 (Aviva systems biology, San Diego, CA), α-smooth muscle actin (α-SMA, Abcam, Cambridge, MA). Next, the section was incubated for 1 h with FITC-labeled goat anti-rabbit IgG secondary antibody (Santa Cruz Biotechnology) or Alexa Fluor goat anti-rabbit IgG H &C secondary antibody. Sections were counterstained with Ultra Cruz Mounting Medium with 4′,6-diamidino-2-phenylindole (DAPI; sc-24941, Santa Cruz Biotechnology) and cover slipped. Fluorescent images were taken using the Nikon Eclipse TE2000-U fluorescence microscope (Nikon Corp., Tokyo, Japan) and a Nikon LWD 0.52 digital camera. Fluorescent intensity was quantified using Image J software.

### Reverse-transcription polymerase chain reaction (RT-PCR)

RT-PCR for transcript levels of kinin B1 receptors in lung and right heart, and atrial natriuretic peptide (ANP), brain natriuretic peptide (BNP), beta myosin heavy chain (βMHC) connective tissue growth factor (CTGF), Collagen I and IV were measured in rat right ventricle as previously described [14, 20].

### Western blot

Western blot experiments were performed to determine protein expression of extracellular signal regulated protein kinase (ERK1/2), phospho-ERK1/2, AKT and phospho-AKT in lung tissues as previously described [21]. Briefly, the lung protein extracts were separated by using SDS-PAGE and transferred to nitrocellulose membranes. The blots were incubated with primary antibodies against ERK1/2, phospho-ERK1/2, Akt, phospho-AKT and β-actin (all from Santa Cruz Biotechnology, Santa Cruz, CA), followed by incubation with HRP-conjugated secondary antibody. Immunoreactivity was detected using an enhanced chemiluminescence Western blotting detection kit (Amersham, Piscataway, NJ). Results were quantified using Image J software.

### Survival study

Additional groups of animals were used to evaluate the effect of BI113823 on long-term survival following monocrotaline-induced PAH in left pneumonectomized rats. Animal preparation was the same as described above for the first series of experiments. Three weeks after the administration of monocrotaline, animals received daily treatments of vehicle or BI113823 (30 mg kg^−1^, p.o., b.i.d.). This experiment was terminated 6 months after monocrotaline injection.

### Human monocyte assay

#### Cell culture and treatment

U937 human monocytic cells (ATCC, Manassas, VA) were grown in RPMI 1640 supplemented with 10% FBS. Cells (1 × 10^6^ cells/well) were seeded in 24-well plates in serum free-RPMI medium and were treated with LPS (100 ng/ml) in the presence or absence of kinin B1 receptor antagonist BI113823 (0.001, 0.01, 0.1, 1 µM). Culture medium were collected at 24 h and stored in aliquots at − 80 °C for biochemical assays. Levels of tumor necrosis factor (TNF)-α (PeproTech, Rocky Hill, NJ, USA), and IL-1β (R&D Systems, Minneapolis, MN, USA) in the culture medium were determined by using enzyme immunoassay kits according to the manufacturer’s instructions. Immunohistochemical analysis for kinin B1 receptor, MMP-2 and MMP-9 expression in human macrophages was measured as described above.

### Cell migration assay

U937 human monocytes were treated with TNF-α (25 ng/ml) in the presence or absence of kinin B1 receptor antagonist BI113823 (0.001, 0.01, 0.1, 1 µM). 1 × 10^6^ treated cells (in 0.5 ml serum free-RPMI medium) were added to each upper chamber of transmigration plate (#140654, Thermo Scientific, Waltham, MA, USA) and 1.5 ml of serum free-RMPI media containing the same concentrations of TNF-α and the BI113823 in upper chambers were added to lower chambers. Cells were incubated at 37 °C and 5% CO_2_ for 24 h. After 24 h incubation, cells transmigrated to lower chambers were collected and counted using hemocytometer.

### Statistical analysis

All data are reported as means ± SEM. Statistical differences were determined by analysis of variance for repeated measures followed by Bonferroni’s post hoc test using GraphPad Prism 5. P values < 0.05 were considered statistically significant differences.

## Results

### Reversal of the progression of PAH and prolong of long term survival

**Fig. 1:**
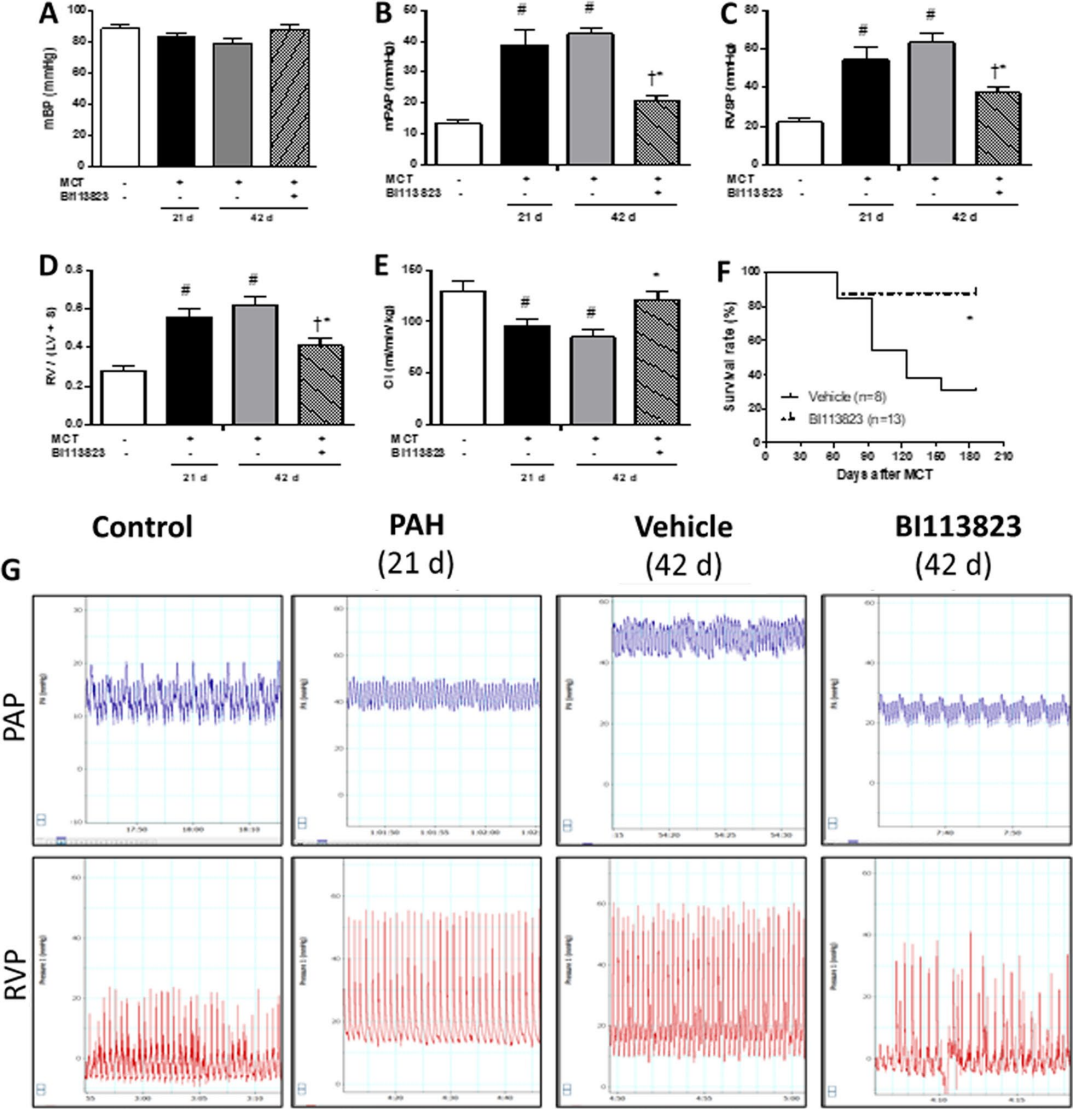
BI113823 reverses the pulmonary arterial hypertension. Measurement of **A** mean blood pressure (mBP), **B** mean pulmonary arterial pressure (mPAP), **C** right ventricular systolic pressure (RVSP), **D** ratio of right ventricular weight to left ventricular plus septum weight [RV/(LV + S)], and **E** cardiac index (CI) in sham control group rats (n = 8), PAH-W3 group rats (pneumonectomized rats at 3 weeks after receiving monocrotaline) (n = 8), vehicle group rats (pneumonectomized rats received monocrotaline and vehicle treatment) (n = 8), and BI113823 group rats (3 weeks of treatment with selective kinin B1 receptor antagonist BI113823 (30 mg/kg, p.o., b.i.d.) starting from 3 weeks after monocrotaline injection in pneumonectomized rats) (n = 8). All values are expressed as mean ± SEM, n = 6–8. ^#^p < 0.05 vs. sham control; ^†^p < 0.05 vs. control (D21); *p < 0.05 vs. vehicle. **F** Survival rates 6 months following administration of monocrotaline in pneumonectomized rats treated with vehicle vs. BI113823. *p < 0.05 versus the vehicle group. **G** Sample tracing of pulmonary arterial pressure and right ventricular pressure from rats

**Fig. 2:**
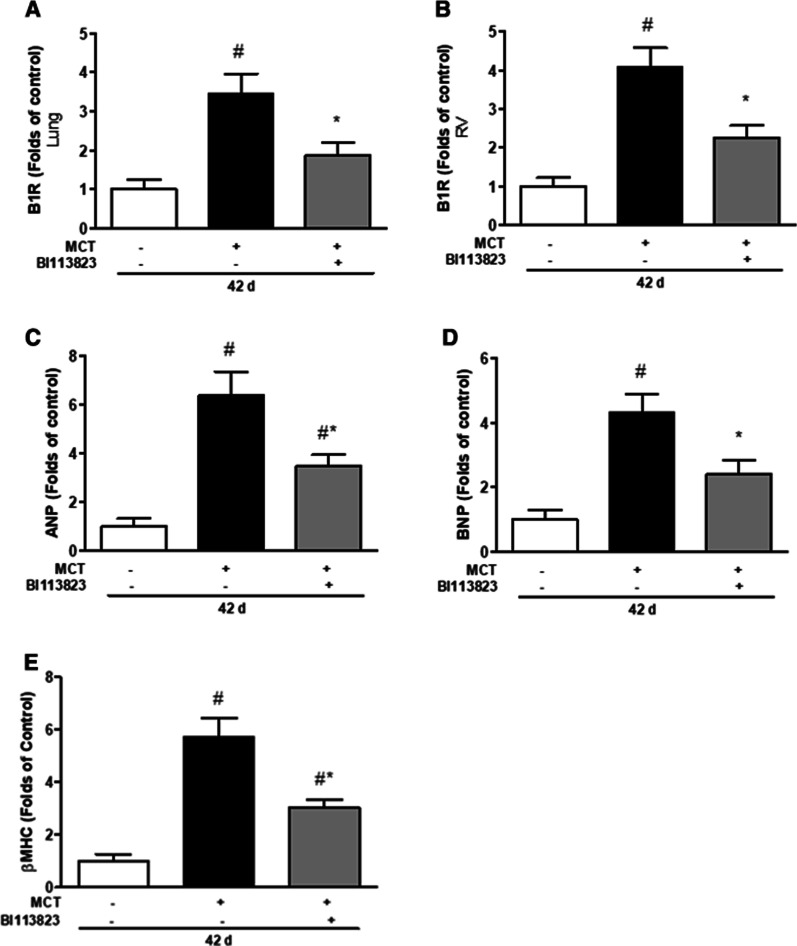
BI113823 attenuates cardiac expression of mediators. Expression of **A** B1R (in lung), **B** B1R, **C** ANP, **D** BNP, **E** βMHC mRNA in rat right ventricles at 42 days after MCT injection. All values are expressed as mean ± SEM, n = 6. ^#^p < 0.05 vs. sham control; *p < 0.05 vs. vehicle

Severe PAH, vascular remodeling and right heart hypertrophy was present at 3 weeks after MCT injection in left pneumonectomized rats [14]. Late treatment with BI113823 reversed the progression of pulmonary arterial hypertension, prevented the transition from PAH to right heart failure and prolonged long term survival. Arterial blood pressure was not significantly different among all study groups. BI113823 therapy reduced PAP (↓ by 50%), RVP (↓ by 41%), and RV/(LV + S) ratio (0.62 in BI113823 group vs. 0.41 in vehicle group), and improved CI (↑ by 42%) compared to vehicle treated animals (Fig. 1A–E, G). Furthermore, PAH led to the death of 69% (9/13) in the vehicle-treated animals, compared to 13% (1/8) in BI113823-treated animals at 6 months after MCT injection in left pneumonectomized rats (Fig. 1F). The mRNA expression of kinin B1 receptor in lung and right heart was marked upregulated in lung and right heart in vehicle treated PAH rats, and was reduced in rats treated with BI113823 (Fig. 2A, B). The mRNA expression of ANP, BNP and β-MHC in right heart was reduced in BI113823 treated rats compared to vehicle control (Fig. 2C–E).

### Reversal of the progression of tissue fibrosis and pulmonary vascular remodeling

**Fig. 3:**
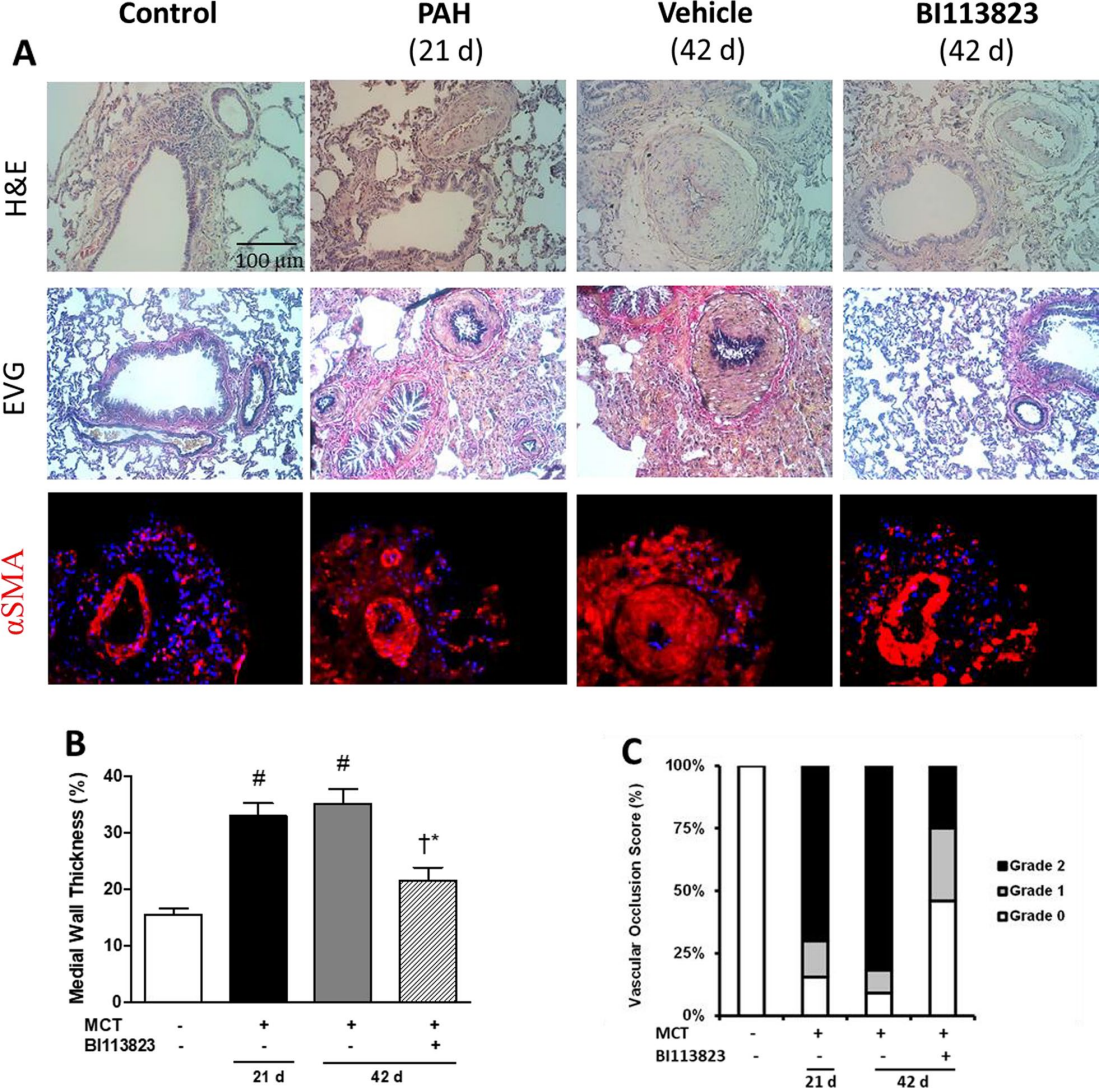
BI113823 reverses the pulmonary arterial neointimal formation. **A** Histological findings of the pulmonary arteries. Top, hematoxylin and eosin (HE) staining; middle, Elastic van gieson (EVG); bottom, immunohistochemical staining of ɑSMA (red color), **B** medial wall thickness of pulmonary arteries, **C** vascular occlusion score of pulmonary arteries in each treatment group. Blue color: DAPI staining of cell nuclei in tissue. All values are mean ± SEM, n = 6–8. ^#^p < 0.05 vs. sham control, ^†^p < 0.05 vs. control (D21); *p < 0.05 vs. vehicle

**Fig. 4:**
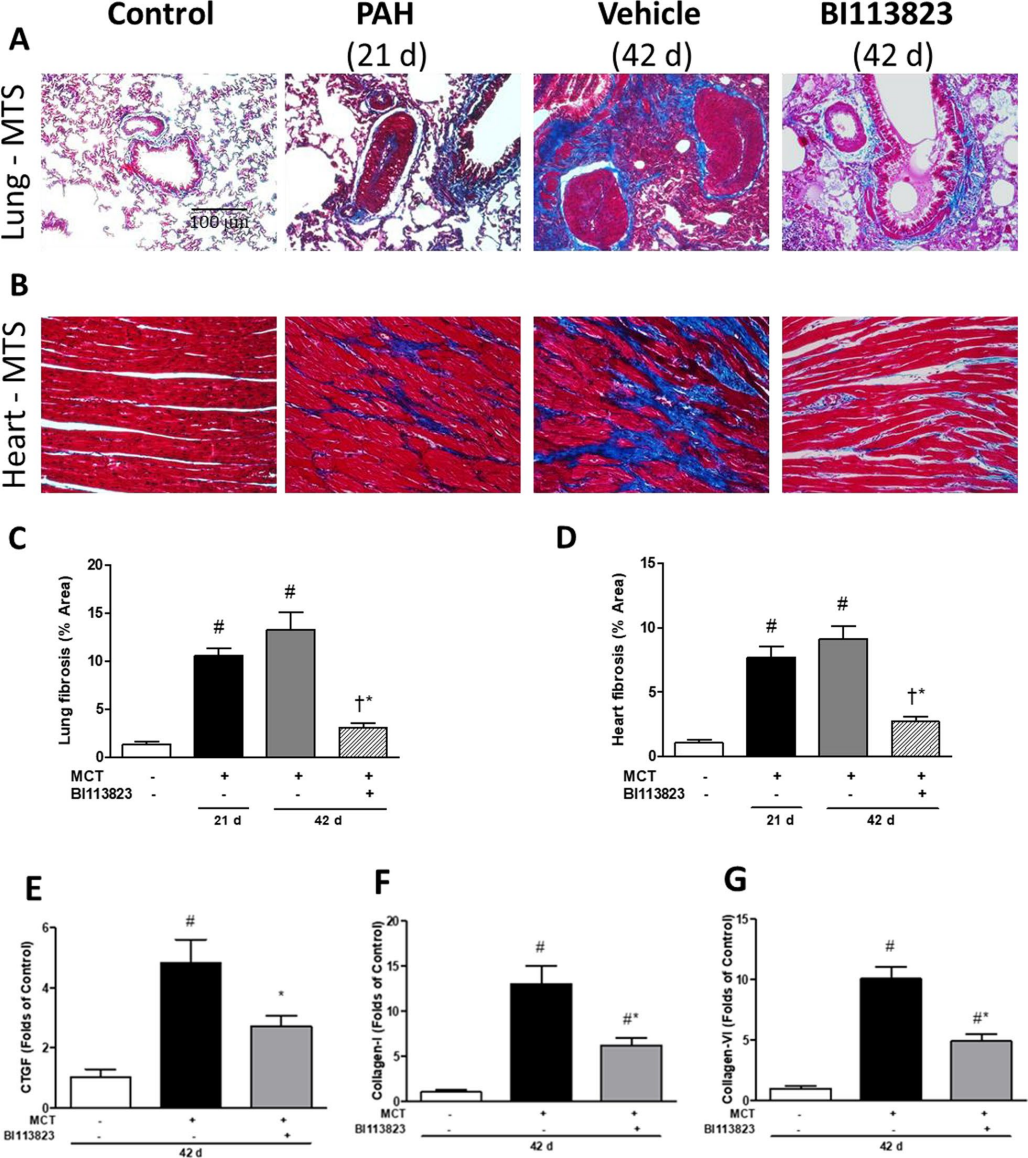
BI113823 reduces fibrosis in lung and heart in pulmonary arterial hypertension. **A**, **B** Masson trichrome staining for collagen (MTS) (blue color) in lung and heart, and their quantification. **C** Lung fibrosis, and **D** right ventricular fibrosis in each treatment group. Fibrotic gene expression of **E** CTGF, **F** Collagen I and **G** Collagen IV in lungs. All values are mean ± SEM, n = 6–8. ^#^p < 0.05 vs. sham control, ^†^p < 0.05 vs. control (D21); *p < 0.05 vs. vehicle

BI113823 therapy also reversed the adverse changes in the lung and heart remodeling (Fig. 3A). BI113823 treated animals had a significant decrease in medial wall thickness (↓ by 41% vs. vehicle control) and in vascular occlusion scores (Grade I and II occlusion: 29% and 25% vs. 9% and 81% in vehicle control) (Fig. 3B, C). Furthermore, BI113823 therapy also prevented the progression of fibrosis in lung and right heart (Fig. 4A–D) and the mRNA expression of CTGF, collagen I and IV was significantly reduced in BI113823 treated rats compared to vehicle treated rats (Fig. 4E–G).

### Reduction of inflammatory cell infiltration and cytokine production

**Fig. 5:**
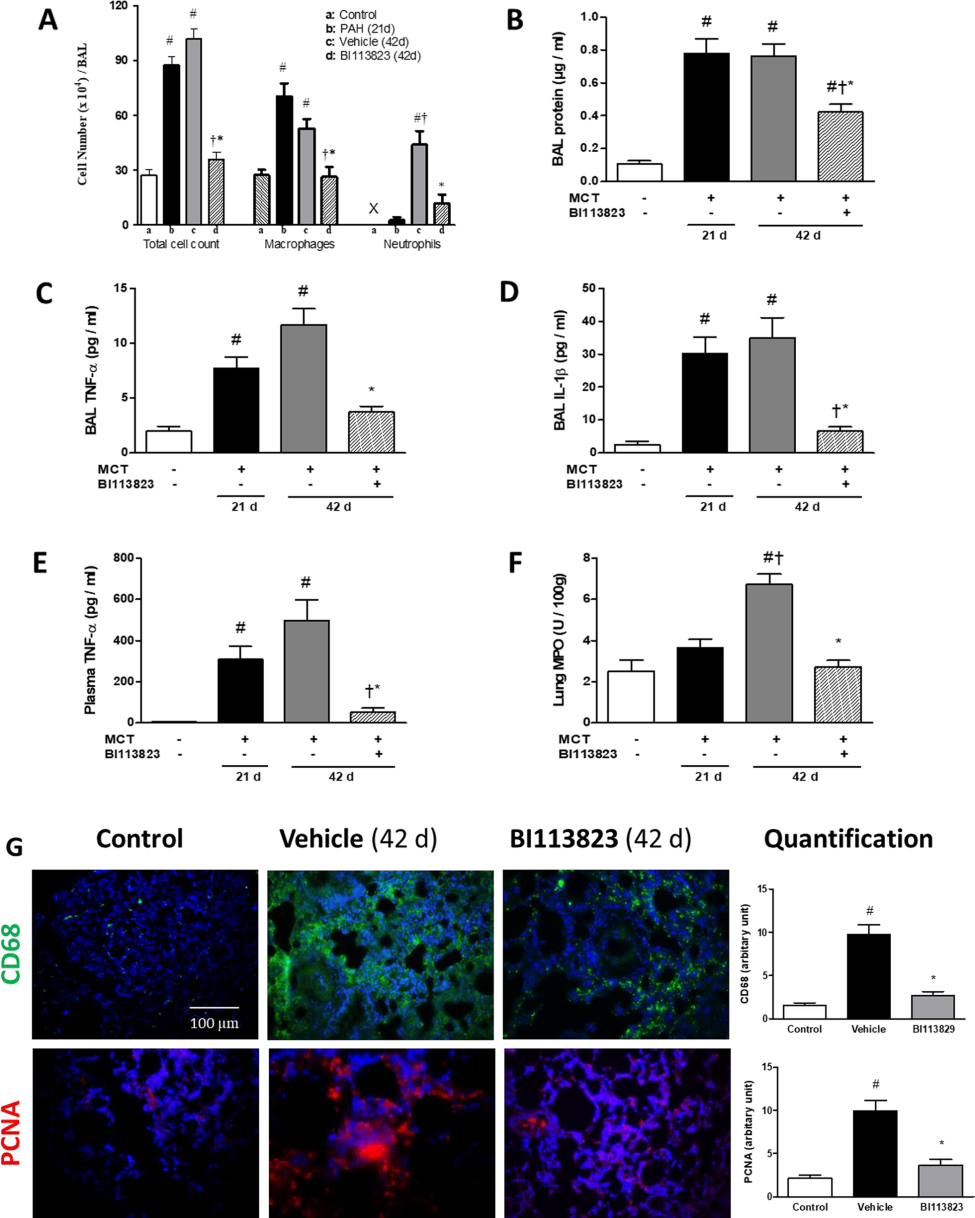
BI113823 inhibits inflammatory cell recruitment and cytokines production in an experimental model of PAH- induced by monocrotaline injection in pneumonectomized rats. **A** Inflammatory cell count in bronchoalveolar lavage (BAL), **B** protein content, **C** TNF-ɑ, and **D** IL-1β in BAL, **E** TNF-α in plasma, and **F** Lung MPO activity of each treatment group. Immunohistochemical analysis for **G** CD-68 (green color) and proliferating cell nuclear antigen (PCNA) (red color) and their quantification in lung sections from each treatment group. Blue color: DAPI staining of cell nuclei in tissue. All values are mean ± SEM, n = 6–8. ^#^p < 0.05 vs. sham control; ^†^p < 0.05 vs. control (D21); *p < 0.05 vs. vehicle

Macrophage cell recruitment is a key feature in the development of pulmonary artery hypertension [4, 14]. Consistent with previous findings, bronchoalveolar lavage samples from MCT-challenged animals showed that macrophages are the majority of inflammatory cell influx at 3 weeks after MCT challenge. However, at 6 weeks following MCT-challenge, in addition to macrophage influx, there was also an increase in the number of neutrophils influx in the airway. In contrast, in rats treated with BI113823, the number of macrophages and neutrophils in BAL were reduced by 48% and 80% compared to the vehicle-control animals, respectively (Fig. 5A). Furthermore, BAL protein content, levels of IL-1β, and TNF-α in lavage or plasma, and lung MPO activity were significantly lower in rats treated with BI113823 compared to vehicle controls (Fig. 5B–F). In addition, in vehicle-treated, MCT-injured lung tissues, there was marked recruitment of CD-68 positive macrophages into perivascular areas (Fig. 5G). There was also substantial vascular cell proliferation in the thickened media layer of the PA, as evidenced by a marked increase of PCNA-positive cells in vehicle-treated animals (Fig. 5G). In contrast, treatment with BI113823 significantly reduced the perivascular macrophage accumulation and vascular cell proliferation (Fig. 5G).

### Signaling pathways in lung

**Fig. 6:**
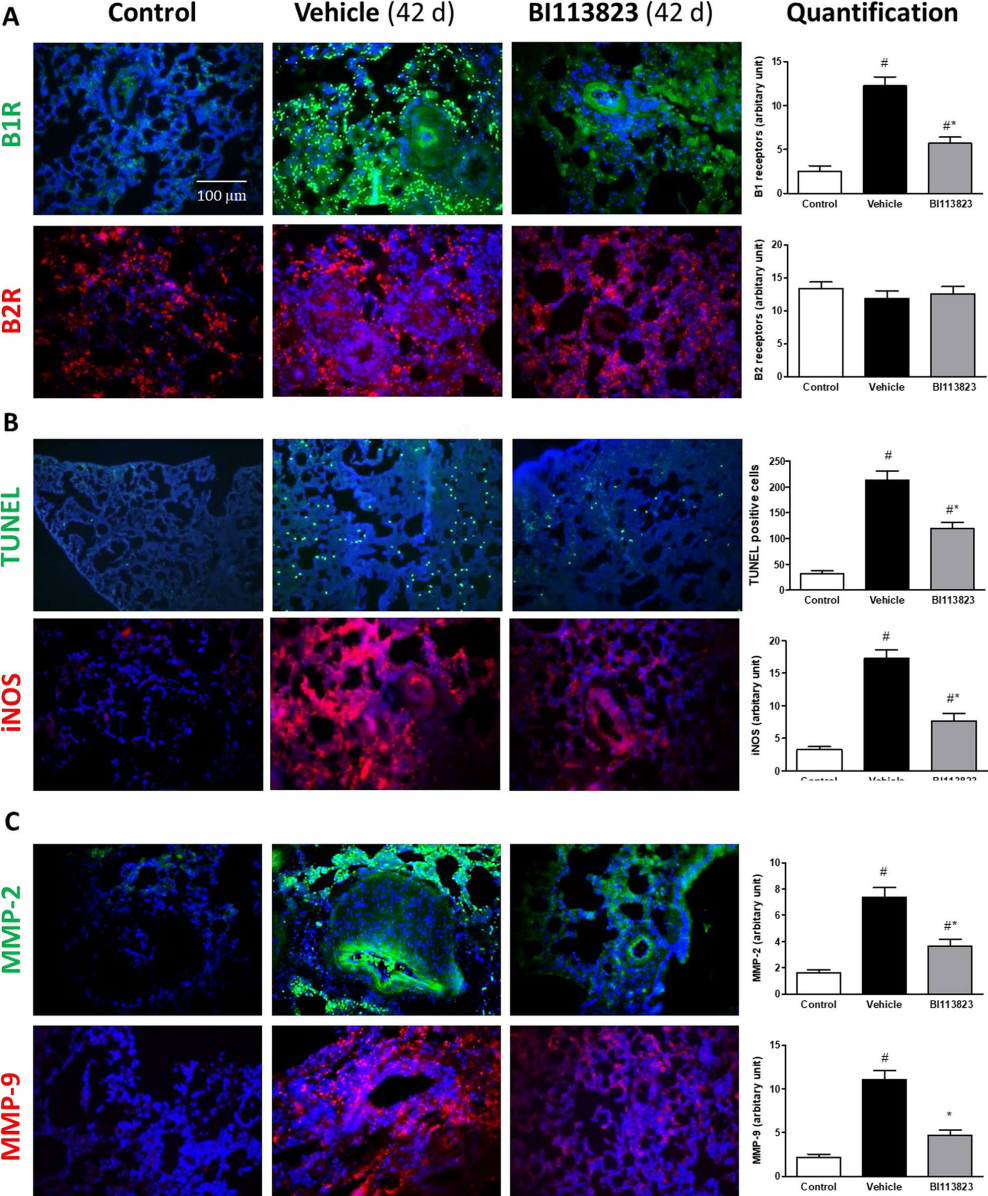
Immunohistochemical analysis for **A** kinin B1 (green color) and B2 receptors (red color), **B** Apoptotic cells (green color) and iNOS (red color) expression and **C** matrix metalloproteinase (MMP)-2 (green color) and MMP-9 (red color) in lung sections from each treatment group. Blue color: DAPI staining of cell nuclei in tissue. All values are mean ± SEM, n = 6. ^#^p < 0.05 vs. sham control, *p < 0.05 vs. vehicle

**Fig. 7:**
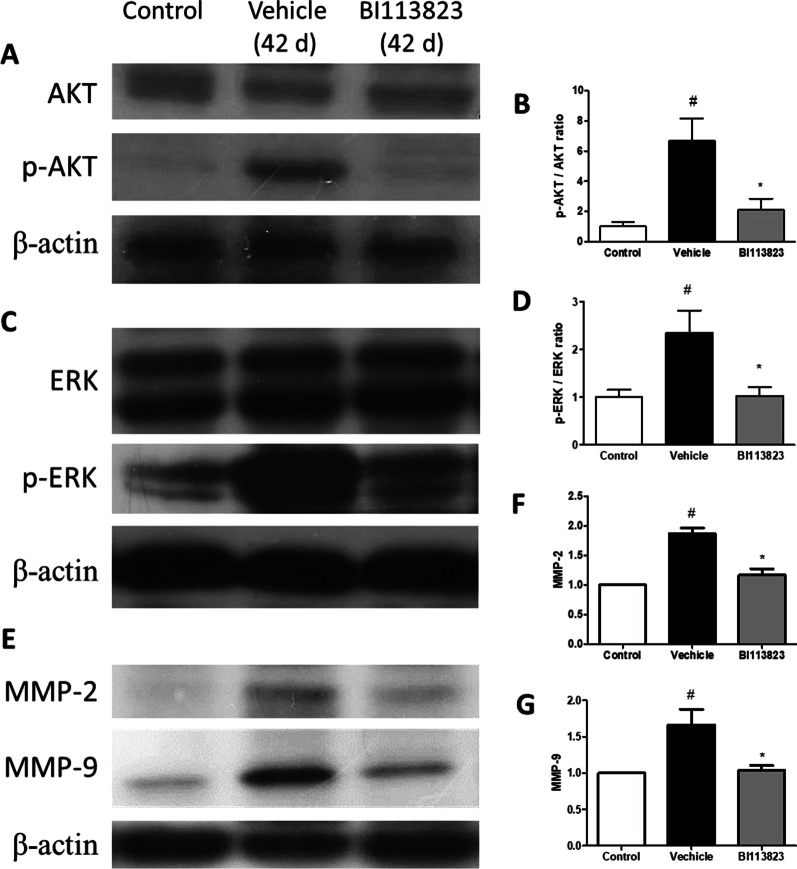
Western blots of the expression of p-ERK/ERK, p-AKT/AKT, MMP)-2, MMP-9 and β-actin in lung tissues. **A**, **C**, **E** Representative immunoblots of p-ERK/ERK, p-AKT/AKT, MMP)-2, MMP-9 and β-actin. **B**, **D**, **F**, **G** Mean densitometric analysis of immunoblots. All values are mean ± SEM. n = 5–6. ^#^p < 0.05 vs. sham control, *p < 0.05 vs. vehicle

Kinin B2 receptors were expressed constitutively in rat lung (Fig. 6A). There was no significant difference of B2 receptor expression among study groups (Fig. 6A). Kinin B1 receptors were weakly expressed in rat lung in control animals, but were markedly upregulated in vehicle-treated MCT-injured lung tissues (Fig. 6A). In contrast, B1 receptor expression was significantly reduced in animals treated with BI113823 (Fig. 6A). These findings confirm that kinin B1 receptors play an important role in the pathogenesis and progression of pulmonary hypertension. Furthermore, tunnel positive cells, protein levels of inducible nitric oxide synthase (iNOS), MMP-2 and MMP-9 were also greatly increased in vehicle-treated MCT-injured lung tissues. Treatment with BI113823 significantly decreased tunnel positive cells and the expression of iNOS, MMP-2 and MMP-9 (Fig. 6B, C). In addition, we measured the AKT, ERK1/2, MMP-2 and MMP-9 expression and phosphorylation in lung tissues by Western blotting (Fig. 7A–G). No significant difference in AKT and total ERK1/2 expression was observed among study groups. However, there were significant increases in AKT and ERK1/2 phosphorylation in vehicle-treated MCT-injured lung tissues. Treatment with BI113823 significantly reduced AKT & ERK1/2 phosphorylation and MMP-2 & MMP-9 expression compared to vehicle-treated controls (Fig. 7A–G).

### Reduction of TNF-α production, MMPs expression and cell migration in human monocytes

**Fig. 8:**
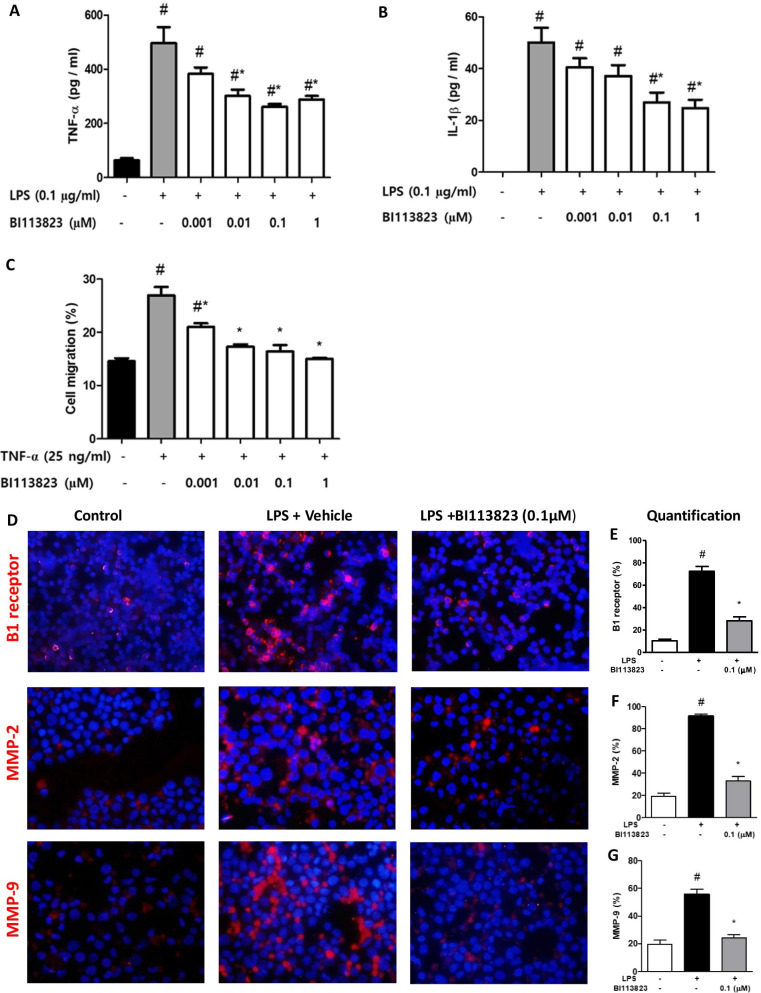
Effects of kinin B1 receptor antagonist BI113823 on LPS-induced **A** TNF-α and **B** IL-1β production, **C** TNF-α-induced monocyte migration, and **D**–**G** LPS-induced expression of B1 receptors (red color), MMP-2 (red color) and MMP-9 (red color) in U937 human monocytes. Blue color: DAPI staining of cell nuclei. Data are mean ± SEM, n = 5–6, ^#^p < 0.05 vs. control, *p < 0.05 vs. vehicle

Macrophage recruitment is highly implicated in the pathogenesis of PAH [6, 7]. We determined the effects of kinin B1 receptor mediated inflammatory responses in human monocytes. Treatment with BI113823 (1 nM to 1 µM) significantly reduced LPS-induced production of TNF-α and IL-1β, and inhibited TNF-α induced monocyte migration (Fig. 8A–C). Furthermore, LPS-induced increase in the expression of kinin B1 receptors, MMP-2 and MMP-9 were inhibited by the treatment with BI113823 (Fig. 8D–G).

## Discussion

The present study demonstrates that kinin B1 receptor blockade with BI113823 reverses pre-existing pulmonary arterial hypertension, cardiovascular remodeling, and prevents right heart failure and death. The effects afforded by BI113823 appears to be related to inhibition of macrophage infiltration, and thus subsequently down-regulation of proinflammatory mediators, including cytokines, matrix metalloproteinase proteins, as well as inhibition of ERK and PI3K/Akt signaling pathways.

### Reversing advanced PAH and preventing right heart failure and death

In the present study, the rats received two “hits” combination of pneumonectomy and MCT administration developed severe pulmonary hypertension and vascular remodeling that resemble those seen in humans with PAH [14, 18, 19]. Three weeks following MCT administration in pneumonectomized rats, pulmonary hypertension and pulmonary vascular neointimal formation were evident. Early treatment with BI113823 (week 1–3 following MCT injection) protected lungs from developing pulmonary arterial hypertension and vascular remodeling [14]. BI113823 treatment (week 4–6 following MCT injection), reversed the progress of vascular remodeling with neointimal formation, lessened pulmonary hypertension and right ventricular hypertrophy, and improved long term survival.

Sustained vasoconstriction and vascular remodeling with neointimal formation are major factors that contribute to the complex pathogenesis of PAH [1, 2]. Vasoconstriction and progressive narrowing of small pulmonary arteries and arterioles result in increased pulmonary vascular resistance for blood flow through the lungs, leading to elevated blood pressure in the pulmonary circulation and right ventricular pressure overload [1, 2, 18]. Kinin B1 receptors mediate strong, endothelium-independent vascular constrictions in arteries following injury, but have no response in normal arteries [19, 22]. Kinin B1 receptors have been shown to mediate strong contractile responses in pulmonary arteries of piglets with sepsis and in endotoxin-damaged pig coronary arteries, and decrease epicardial diameters in heart transplant patients [19, 22]. These contractile responses in cardio-pulmonary vascular beds to kinin B1 receptor agonist des-Arg9-BK are mediated by coupling to cyclooxygenases-2 and activation of thromboxane-prostanoid receptors [19, 22]. Kinin B1 receptors up-regulation in vascular smooth muscle cells can be induced by various mediators, including cytokines, endotoxin, angiotensin II and endothelin-1, and may therefore subsequently increase cardio-pulmonary vascular tone [11, 19, 22].

In PAH, death generally occurs due to right heart failure [1, 2]. Kinin B1 receptors are also involved in the pathogenesis of cardiac dysfunction [23, 24]. Upregulation of B1 receptors was seen within hours, days and weeks after myocardial injury [20, 25, 26]. Kinin B1 receptor deletion attenuated diabetic cardiomyopathy with improved systolic and diastolic function in comparison with diabetic control mice [24]. Treatment with BI113823 attenuated postinfarction cardiac hypertrophy and improved cardiac performance following prolonged myocardial infarction in rats [25]. In the present study, treatment with BI113823 rescued preexisting advanced PAH, restored myocardial function, and improved long term survival. Collectively, the protective effects afforded by BI113823 were attributed to regulating pulmonary vascular tone, attenuating cardiovascular remodeling and improving cardiac function.

### Macrophages and cardiovascular remodeling in PAH

Animal experiments and clinical human studies indicate that inflammation contributes to the development of PAH [2–7]. A prominent pathological feature of PAH is perivascular accumulation of macrophages [1, 2]. CD68 positive macrophages are prominent in advanced plexiform lesions observed in experimental and clinical PAH [2–7]. Macrophage accumulation in the aortic wall during angiotensin II infusion in mice is associated with fibrosis, elastin loss, and elevated blood pressure [27]. Activation of macrophages is also closely linked to proliferation of vascular fibroblasts in patients and in experimental models of PAH [28]. Macrophage depletion lowers blood pressure and restores sympathetic nerve adrenergic receptor function in mesenteric arteries of DOCA-salt hypertensive rats [29]. Macrophage depletion also prevents portopulmonary hypertension in an experimental animal model of hepatopulmonary syndrome in rats [30]. Blocking macrophage derived leukotriene B4 also prevents endothelial injury and reverses pulmonary hypertension [4]. These findings demonstrate that perivascular macrophage accumulation plays a key role in pulmonary vascular remodeling and development of PAH [2–7, 27–30].

Kinin B1 receptors play an important role in inflammatory cell activation, and mediate diverse pathological processes by triggering the release of various mediators including cytokines, prostaglandins, leukotrienes, and reactive oxygen species [9, 10]. Kinins stimulate bovine alveolar macrophages to activate the chemotaxis of neutrophils, monocytes, and eosinophils through B1 receptor mediated responses [31]. In patients with atheromatous disease, there is high kinin B1 receptor expression, but low kinin B2 receptor expression on foamy macrophages within thickened intimal plaques [15]. In the present study, B1 receptor expression was upregulated in the lung tissues of MCT-challenged pneumonectomized rats in vivo and in LPS treated human monocytes in vitro. Treatment with kinin B1 receptor antagonist BI113823 decreased MCT challenge-induced up-regulation of kinin B1 receptors, recruitment of macrophages and neutrophils in the lungs and inhibited TNF-α induced human monocyte migration. There was also a significant reduction of CD-68 positive macrophages into perivascular areas in rats treated with BI113823 compared to vehicle-control animals. Furthermore, the reduction in macrophage infiltration was accompanied by a significant reduction in proinflammatory cytokine TNF-α, IL-1β release in bronchoalveolar lavage and plasma, as well as in cultured human monocytes. Cytokines such as TNF-α and IL-1β are major contributing factors in the pathogenesis of PAH [32, 33]. These findings suggest that inhibition of macrophage recruitment and cytokine production are important contributing factors to the attenuation of pulmonary arterial hypertension and vascular remodeling afforded by B1 receptor inhibition with BI113823. The reduction of the expression of kinin B1 receptors in BI113823 treated animals may be associated with its effects in inhibition of inflammatory mediators and pathways.

### Role of cellular pathways

The process of pulmonary vascular remodeling and neointimal formation is attributed to pathological changes to cell signaling pathways in PAH [1–3]. Pulmonary hypertension has been shown to activate cell signaling pathways such as the mitogen-activated protein kinases (MAPKs), and phosphatidylinositol 3-kinase (PI3K)/serine-threonine kinase AKT signaling pathways [1–3, 34]. AKT and ERK are parallel signaling pathways activated by Growth factors & Mitogens, both phosphorylates tuberous sclerosis complex 2 (TSC2) to suppress the inhibitory effect of the TSC1–TSC2 complex on mTORC1, thus leading to increased mTORC1 signaling which phosphorylates eukaryotic initiation factor 4E-binding protein (4E-BP) and p70S6K [35].

Both AKT and ERK signaling pathways can be activated by growth factors, such as PDGF in vascular smooth muscle cells, regulating cell cycle progression and cell survival [35, 36]. The PI3K-Akt and MEK/ERK signaling pathway are also activated rapidly in response to TLR activation in macrophages to regulate the inflammatory stimuli, migration and phagocytosis [37].

The PI3K-Akt and MEK/ERK signaling pathway are involved in cell proliferation, survival, extracellular remodeling and fibrosis which are major features of pulmonary vascular smooth muscle cells associated with PAH [38–40]. AKT is an essential downstream target of PI3K for the remodeling of actin filaments to increase cell migration and for proliferation of VSMCs involved in vascular remodeling [39, 40]. The MAPKs regulate diverse cellular programs by relaying extracellular signals to intracellular responses [41]. Activation of MAPK promotes cellular growth and proliferation through the phosphorylation and inactivation of pro-apoptotic proteins, and that contributes to the hypoxia-induced PAH-associated pulmonary vascular remodeling [41, 42]. Phosphorylation of ERK1/2 is significantly increased in MCT induced PAH rat model and Inhibition of the ERK1/2 signaling pathway activation prevents pulmonary vascular remodeling, elevated right ventricular pressure and improves right ventricular hypertrophy in experimental animal models of PAH model [43, 44]. Pro-inflammatory mediators such as TNF-α and interleukins induce kinin B1 receptor upregulation by activate intracellular MAPK/NF-κB and their related signal pathways including iNOS and PI3K/AKT signaling pathways [12, 45]. Kinin B1 receptor stimulation triggers synthesis and secretion of matrix metalloproteases (MMPs), and subsequent phosphorylation of ERK1/2 and PI3K/AKT [44]. The MMPs play a crucial role in tissue remodeling through the regulation of extracellular matrix degradation, cell migration, differentiation, and cell proliferation [46]. In the present study, kinin B1 receptor blockade with BI113823 significantly reduced the expression of matrix metalloproteinase (MMP)-2, MMP-9 and iNOS, and inhibited the phosphorylation of ERK1/2 and AKT, and compared to measurements in vehicle-treated rats. In human monocytes, BI113823 reduced cytokine production, inhibited monocyte migration, and down-regulated the expression of MMP-2 and MMP-9. Cytokines, including TNF-α and IL-1, have been shown to regulate the production of macrophage-derived MMPs in arthrosclerosis lesions [47].

It has been reported that compounds (e.g., simvastatin) reduces pulmonary hypertension by inducing apoptosis of neointimal smooth muscle cells [19]. However, our study show that BI113823 attenuates growth factors and hypoxia stimulated pulmonary artery smooth muscle cell migration and proliferation [14], does not induce apoptosis in this study.

Kinin peptides enhance inflammatory and oxidative responses promoting apoptosis in a Parkinson’s disease cellular model [48]. In a rat model of sepsis via CLP, BI113823 reduced inflammation and cell apoptosis in liver tissue [13]. In this study, BI113823 treatment significantly reduced inflammatory cell infiltration, inflammatory markers like TNFα, IL-1β, B1R, MPO, as well as apoptotic cells in lung tissue which suggest reduced tissue inflammatory injury.

Collectively, the findings of the present study suggest that inhibition of macrophage recruitment, down regulation of cytokine production and MMPs, as well as down-stream pathways by BI113823 may contributed to the reversal of the progression of pulmonary hypertension and vascular remodeling.

## Conclusion and perspectives

We conclude that BI113823 reverses the progression of vascular remodeling, lessens pulmonary arterial hypertension, and prevents right heart failure and death. The protective effects afforded by BI113823 are mediated through inhibition macrophage infiltration, and subsequently down-regulation of proinflammatory mediators. This regimen offers a unique novel approach for anti-inflammatory and remodeling therapy in progressed pulmonary hypertension.

## Data Availability

The datasets used and /or analyzed during the current study are available from the corresponding author on reasonable request.

## Abbreviations

PAH: Pulmonary arterial hypertension
mPAP: Mean pulmonary arterial pressure
RVSP: Right ventricular systolic blood pressure
PAP: Pulmonary arterial pressure
PCNA: Proliferating cell nuclear antigen
MMP: Matrix metalloproteinase
MIP-1α: Macrophage inflammatory protein-1 alpha
MCT: Monocrotaline
BAL: Bronchoalveolar lavage
TNF-α: Tumor necrosis factor alpha
MPO: Myeloperoxidase
α-SMA: α-Smooth muscle actin
MAPKs: Mitogen-activated protein kinases
PI3K: Phosphatidylinositol 3-kinase
